# Research on the Mechanical Durability Performance and Action Mechanism of Basalt Fiber-Reinforced Concrete for Ship Lock Wall

**DOI:** 10.3390/polym18131587

**Published:** 2026-06-26

**Authors:** Benkun Lu, Jie Chen, Shuncheng Xiang, Zhe Peng, Changyu Liu, Haotian Yu, Yasi Ye

**Affiliations:** 1 School of Hydraulic and Ocean Engineering, Changsha University of Science & Technology, Changsha 410114, China; wy769521218@163.com (B.L.); xsc313@hnu.edu.cn (S.X.); 15886738982@163.com (H.Y.); 2 Hunan Provincial Department of Transportation, Changsha 410000, China; 3 Key Laboratory of Water-Sediment Sciences and Water Disaster Prevention of Hunan Province, Changsha 410114, China; 4 Hunan Provincial Water Transport Construction & Investment Group Co., Ltd., Changsha 410029, China; 18674464582@163.com (Z.P.); 13937428202@163.com (C.L.); 5 Hunan Provincial Communications Planning, Survey & Design Institute Co., Ltd., Changsha 410000, China; wyyx1673449@163.com

**Keywords:** basalt fiber concrete, working performance, mechanical properties, early crack resistance, microstructure

## Abstract

To address early-age cracking in concrete walls of hydraulic structures such as ship locks, basalt fibers (BFs) were incorporated as a reinforcement strategy. The effects of varying BF dosages and lengths on the workability, mechanical strength, and crack resistance of concrete were systematically evaluated. Furthermore, the internal microstructure was examined using scanning electron microscopy (SEM), and the durability performance, including impermeability, freeze–thaw resistance, and abrasion resistance, was assessed. The results indicate that workability decreased with increasing fiber content and length. The highest mechanical performance among tested mixes was achieved with 0.1% BF of 9 mm length, increasing 7-day and 28-day compressive strength by 17.47% and 22.59%, respectively, compared to plain concrete. The greatest crack resistance was observed with 0.2% BF of 18 mm length, delaying cracking by 150% and reducing crack width by 85%. Durability tests showed that a 0.2%-18 mm BF mix reduced water permeability depth by 47.37% and a 0.3% BF content optimized abrasion resistance. Freeze–thaw cycles indicated that a 0.3% fiber content effectively maintained the relative dynamic elastic modulus. SEM analysis revealed that BFs act as micro-bridges within the matrix, optimizing pore structure, inhibiting micro-crack propagation, and enhancing concrete density. This study evaluates BF-reinforced concrete and provides a practical reference for improving crack resistance and long-term durability in ship lock structures.

## 1. Introduction

Lock gates are critical components of inland waterways, subjected to long-term forces, including water pressure, earth pressure, ship impact, and harsh environmental conditions such as wet–dry and freeze–thaw cycles. Cracking in lock gate concrete walls, particularly early-age cracking, poses a significant hidden risk that compromises the functionality and durability of ship locks [1,2]. With the advancement of composite materials, basalt fiber-reinforced concrete has emerged as a highly promising material due to its excellent corrosion and crack resistance. It has been shown to significantly improve toughness, impact resistance, fatigue resistance, and shrinkage reduction [3]. Its excellent ability to resist and prevent cracking is especially useful for controlling early-age cracking in concrete [4,5,6,7].

Despite these promising attributes, a critical review of the existing literature reveals several notable gaps. First, a consensus on the optimal fiber parameters remains elusive. For instance, Rama [8] reported that the optimal basalt fiber dosage for tensile strength was 0.35%, with compressive strength generally improving with fiber addition. In contrast, Li [9] found the optimal dosages for compressive and flexural strength to be 1.5 kg/m^3^ (approximately 0.06% by volume), while the optimal dosage for tensile strength was 1.0 kg/m^3^, demonstrating significant variability in reported optimal values. Second, most studies have focused on isolated performance metrics. While Huo [10] and Li [9] explored mechanical properties alongside impermeability, and Guan [11] investigated frost resistance, a systematic evaluation that simultaneously considers mechanical strength, early-age crack resistance, impermeability, freeze–thaw resistance, and abrasion resistance—all of which are critical for the multi-hazard environment of ship lock walls—is conspicuously absent. Third, the underlying reinforcement mechanisms are often superficially addressed. Although some studies [4,5,6,7,12,13] attribute performance improvements to the bridging effect of fibers, a detailed microstructural analysis linking specific fiber parameters (length and dosage) to the observed multi-performance enhancements, particularly in the context of hydraulic concrete, is insufficient.

To address these identified gaps, basalt fiber-reinforced concrete (BFRC) was evaluated for the demanding service conditions of ship lock walls. The main features of this study are as follows:

1. Simultaneous evaluation of multiple properties: This study investigates the coupled effects of fiber length (6–24 mm) and dosage (0.1–0.4%) on workability, compressive strength, early-age crack resistance, impermeability, freeze–thaw resistance, and abrasion resistance. Such an evaluation provides a practical reference for material design in complex hydraulic environments.
2. Engineering-specific parameter evaluation: This study focuses on ship lock applications, where simultaneous resistance to cracking, water penetration, frost damage, and abrasion is important. The recommended fiber parameters are based on test results under these combined considerations.
3. Microstructural observation: Scanning electron microscopy (SEM) was used to examine the microstructure of BFRC, including fiber dispersion, interfacial bonding, and pore structure. The observations provide a visual basis for discussing the relationship between fiber parameters and macroscopic performance.
4. Quantified performance indicators: This study reports specific performance values, e.g., a 150% delay in cracking time for the optimal mix, along with corresponding durability metrics, which can serve as references for engineering design.
5. Through this integrated evaluation, the present study offers a practical reference for the design of durable, crack-resistant concrete structures in inland waterway engineering.

It is acknowledged that individual aspects of basalt fiber reinforcement have been studied previously. The contribution of this work lies in the simultaneous measurement of multiple durability indicators under a single experimental framework tailored to ship lock wall conditions, and in the provision of fiber parameter recommendations derived from these combined tests.

## 2. Overview of the Experiment

### 2.1. Raw Material

Hailuo’s P O 42.5 ordinary silicate cement boasts key physical and mechanical properties as per Table 1, meeting GB 175-2023 standards [14].

Common Grade II fly ash, detailed in Table 2, meets the criteria outlined in DL/T 5055-2007 [15] for its use in hydropower concrete.

Crushed limestone (5–20 mm continuous gradation) was used as coarse aggregate, with a crushing index ≤ 8%, clay content ≤ 0.5%, and an apparent density of approximately 2700 kg/m^3^.

Natural river sand with a fineness modulus of 2.6–2.8 (Zone II) was used as fine aggregate, with a clay content ≤ 1.0% and an apparent density of approximately 2650 kg/m^3^.

Add polycarboxylate high-performance water reducer, which has a 20% water reduction rate and a solid content of 20.1%, at a rate of 0.8% to 1.2% of the weight of the cementitious materials.

Haining Anjie Co., Ltd. (Haining, China) makes basalt fiber in sizes 6 mm, 9 mm, 12 mm, 18 mm, and 24 mm, and these sizes are listed in Table 3 along with their main performance details.

Clean tap water from Changsha City.

Analytically pure hydrochloric acid with a concentration of 1 mol/L. KH550.

Deionized water.

### 2.2. BF’s Acid Etching Treatment

The fibers were immersed in deionized water and stirred manually at a consistent rate of approximately 100 rpm for 30 min to remove surface dust. The water was then decanted. Subsequently, the cleaned fibers were fully submerged in a pre-prepared 1 mol/L hydrochloric acid solution with a fiber-to-solution volume ratio of 1:10 at a room temperature of 20 °C for 2 h. After acid treatment, the fibers were quickly transferred to a large volume of deionized water and stirred for 5 min. This washing process was repeated 3–5 times, with fresh deionized water used each time, until the pH of the rinsing water was neutral (pH 7), as verified by pH test paper, ensuring complete removal of the acid solution.

### 2.3. Proportions of Concrete Mix

Drawing on the guidance of JGJ/T 221-2010 [16] and real-world engineering practices, our research delved into the impact of basalt fiber on concrete by considering five distinct fiber contents: 0%, 0.1%, 0.2%, 0.3%, and 0.4%, all based on concrete volume. The foundational concrete mix was crafted to meet C30 grade standards, adhering to DL/T 5330-2015 [17]. This study opted for a water-binder ratio of 0.40, with cement and fly ash comprising 80% and 20% of the binder, respectively. The sand ratio was fixed at 40%, and a water-reducing agent made up 0.8% of the binder. These specifics culminated in the concrete mix proportion detailed in Table 4.

The selection of basalt fiber parameters was based on a combination of preliminary experimental results and findings from the existing literature [8,9,18]. Fiber contents of 0.1%, 0.2%, 0.3%, and 0.4% by volume of concrete were chosen to span the typical range used in BFRC research, aiming to identify the optimal dosage that balances performance improvement with workability. Fiber lengths of 6 mm, 9 mm, 12 mm, 18 mm, and 24 mm were selected to investigate the effect of fiber aspect ratio on mechanical interlocking and bridging capabilities within the concrete matrix. This range covers commonly available commercial sizes and allows for a systematic assessment of the length effect.

### 2.4. Test Method

#### 2.4.1. Performance Testing of the Work

According to GB/T 50080-2016 [19], the slump of the concrete is measured using a slump cylinder (in millimeters).

#### 2.4.2. Compressive Strength Test

Following GB/T 50081-2019 [20], which outlines the test methods for concrete’s physical and mechanical properties, this study employed a hydraulic compression testing machine to assess compressive strength. Loading at a steady 0.5 MPa/s, this study recorded the failure load. Using 150 mm^3^ cube specimens, this study tested three from each group and averaged their results for accuracy.

#### 2.4.3. Permeability Resistance Test

According to the GB/T 50082-2024 [21], specifically the permeability height method in the water penetration test, the specimens were sealed with paraffin, and the permeameter was started with a constant water pressure set to 1.2 MPa. When water penetration appeared on the surface of a specimen, the time was recorded, and the specimen was pressed into the specimen sleeve. After continuous pressurization for 24 h, the specimens were removed, and a compression testing machine was used to split the specimens longitudinally into two halves to determine the average permeability height.

#### 2.4.4. Freezing Resistance Performance Test

In line with the rapid freezing-and-thawing protocol outlined in SLT 352-2020 [22], the experiment proceeded. This study prepared prismatic concrete specimens, each sized 100 mm × 100 mm × 400 mm, and allowed them to cure for 28 days. Four days prior to testing, specimens were submerged in (20 ± 2) °C water to reach full saturation. Subsequently, they were placed in a freeze–thaw machine. Every 25 cycles, this study assessed their relative dynamic elastic modulus and mass loss.

#### 2.4.5. Crack Resistance Test

To assess concrete’s resistance to early-age cracking, this study employed the ring test method outlined in CCES 01-2004 [23], a guide for concrete durability design and construction. Initial strain and temperature were logged, specimens were kept in a controlled temperature and humidity setting, and surfaces were monitored regularly for cracks, noting their timing, count, positions, and widths.

#### 2.4.6. Abrasion Resistance Test

The impact-abrasion test followed the underwater steel ball method outlined in the SL/T 352-2020 [22]. A flat cylindrical specimen, measuring 300 mm in diameter and 100 mm in height, was positioned within the testing machine’s container. Subsequently, a predetermined amount of steel balls and clean water were carefully introduced into the container. The machine was then activated, and the shaft’s rotational speed was precisely set to 1200 r/min, initiating the test. Over a period of 72 h, the steel balls repeatedly struck and abraded the specimen’s surface. Upon completion, the specimen was extracted, dried, and weighed. The specimen’s abrasion resistance strength and wear rate were then determined by analyzing the mass loss caused by abrasion.

#### 2.4.7. SEM Scanning

**Figure 1:**
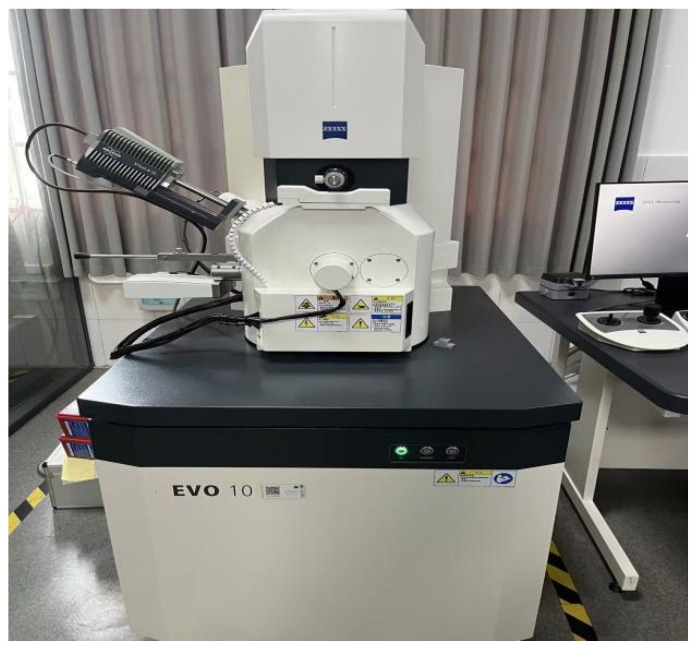
Scanning Electron Microscope.

This study used a high-resolution scanning electron microscope (Figure 1) to look at the inside of basalt fiber-reinforced concrete. After doing some big-scale mechanical tests, this study picked out and gathered the right pieces of concrete, and then ground them down to the right size. Using the scanning electron microscope, we checked the tiny details of the fiber-reinforced concrete at 500 times and 1000 times magnification to understand how basalt fibers make concrete work better.

## 3. Results and Discussion

### 3.1. Working Performance

**Figure 2:**
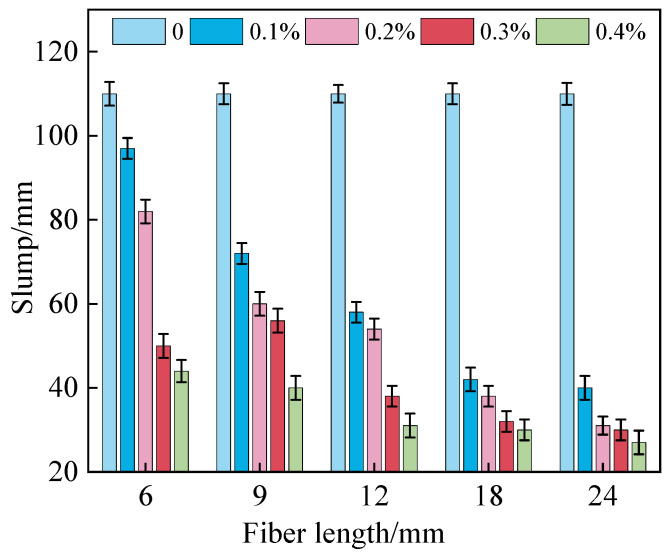
The influence of basalt fiber length and dosage on the slump of concrete.

The slump shows how fluid the concrete is and is a key way to measure how easy the concrete mix is to work with [24]. This study calculated the slump values for the concrete and these are shown in Table 5. Figure 2 shows how the slump changes as the length and amount of basalt fiber in the concrete change.

As shown in Table 5 and Figure 2, the slump of basalt fiber-reinforced concrete decreased with increasing fiber length and content compared to plain concrete. For instance, at a fiber length of 24 mm and a content of 0.4%, the slump decreased by 75%. When the amount of fibers stayed the same, the slump generally got smaller as the fibers got longer. If the fiber content was 0.1%, the slump was 19% lower with 12 mm fibers than with 9 mm fibers, and almost 40% lower than with 6 mm fibers. When the fiber length stayed the same, the slump consistently got smaller as more fibers were added. For 6 mm fibers, the slump was 39% lower with 0.3% fiber content than with 0.2%, and nearly 48% lower than with 0.1%.

Basalt fibers have a small diameter; as fiber length increases, the total surface area also increases. This means more cement paste is needed to cover them when mixed into concrete. Longer fibers also tend to clump together and stick to a lot of cement paste. Longer fibers are also more prone to uneven agglomeration, creating voids and reducing the fluidity and workability of the concrete [18]. When more fibers are added, the random 3D network they form inside the concrete takes up more space. Because basalt fibers bond strongly with cement, the mixture becomes stickier and less runny, reducing its slump [25].

### 3.2. Compressive Strength

**Figure 3:**
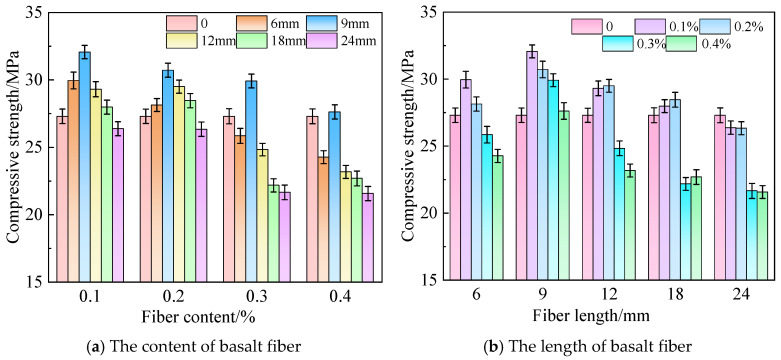
The influence of basalt fiber content and length on the 7-day compressive strength of concrete is illustrated.

The variation in 7-day compressive strength of concrete with basalt fiber content and length is shown in Figure 3.

The compressive strength of concrete without basalt fiber after 7 days was 27.30 MPa. At a fiber content of 0.1%, the compressive strength first increased and then decreased with increasing fiber length. The highest compressive strength, 32.07 MPa, was reached when the fiber was 9 mm long, which was 17.47% higher than that of concrete without basalt fiber. When the basalt fiber content was 0.2%, the compressive strength also first rose and then fell with increasing fiber length. The peak compressive strength was 30.72 MPa at a fiber length of 9 mm, marking a 12.53% improvement over concrete without basalt fiber. When the basalt fiber content was raised to 0.3% and 0.4%, only the concrete with 9 mm fibers showed better compressive strength than concrete without basalt fiber, with strengths of 29.92 MPa and 27.62 MPa respectively. For the other four fiber lengths, the compressive strength was lower than that of concrete without basalt fiber.

When the length of basalt fibers stayed the same, researchers noticed that as more basalt fibers were added to the concrete, its compressive strength first went up and then went down [26]. For basalt fibers that were 6 mm long, the highest compressive strength, 29.96 MPa, was reached when 0.1% fibers were added. This was 9.74% stronger than concrete without basalt fibers. When the fibers were 9 mm long, the best compressive strength was 32.07 MPa with 0.1% fibers, which was 17.47% better than concrete without basalt fibers. For 12 mm-long fibers, the maximum compressive strength was 29.50 MPa with 0.2% fibers, which was 8.06% higher than concrete without basalt fibers, and there was not much difference in strength between 0.1% and 0.2% fiber content. With 18 mm-long fibers, the highest compressive strength was 28.46 MPa at 0.2% fiber content, showing a 4.25% improvement over concrete without basalt fibers. When the fibers were 24 mm long, adding any amount of fibers made the concrete weaker compared to concrete without basalt fibers. Overall, with an increase in either fiber content or length, the 7-day compressive strength initially increased and then decreased. The best result was with 0.1% fibers that were 9 mm long, which made the concrete 17.47% stronger than regular concrete.

**Figure 4:**
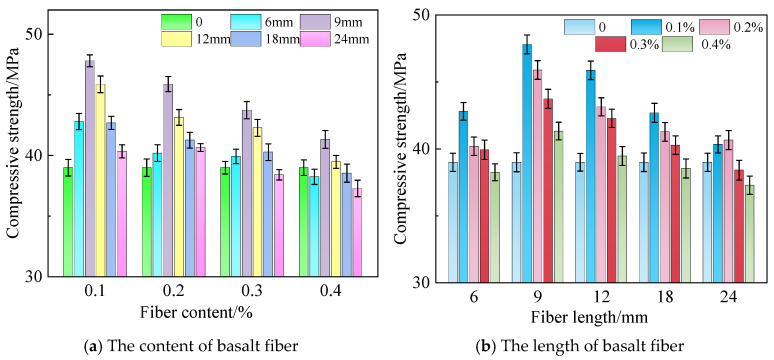
The influence of basalt fiber content and length on the 28-day compressive strength of concrete is illustrated.

The variation in 28-day compressive strength of concrete with basalt fiber content and length is shown in Figure 4.

The compressive strength of concrete without basalt fiber after 28 days was 39.00 MPa. When basalt fiber was added at 0.1% and 0.2%, the compressive strength initially went up and then went down as the fiber length increased, peaking at 47.81 MPa and 45.89 MPa respectively when the fiber length was 9 mm. These peaks were 22.59% and 17.67% higher than the strength of concrete without basalt fiber. When the amount of basalt fiber was raised to 0.3% and 0.4%, the highest compressive strengths were also reached at a fiber length of 9 mm, with values of 43.74 MPa and 41.33 MPa respectively, which were 12.15% and 5.97% better than the concrete without basalt fiber.

When the length of basalt fibers stayed the same, the concrete’s compressive strength initially went up and then went down as more basalt fibers were added, reaching its peak at a 0.1% fiber content. Generally, as the amount or length of basalt fibers increased, the concrete’s 28-day compressive strength also first rose and then fell. The highest compressive strength recorded was 47.81 MPa, achieved with 0.1% basalt fiber content and fibers that were 9 mm long, which is a 22.59% improvement over regular concrete.

**Figure 5:**
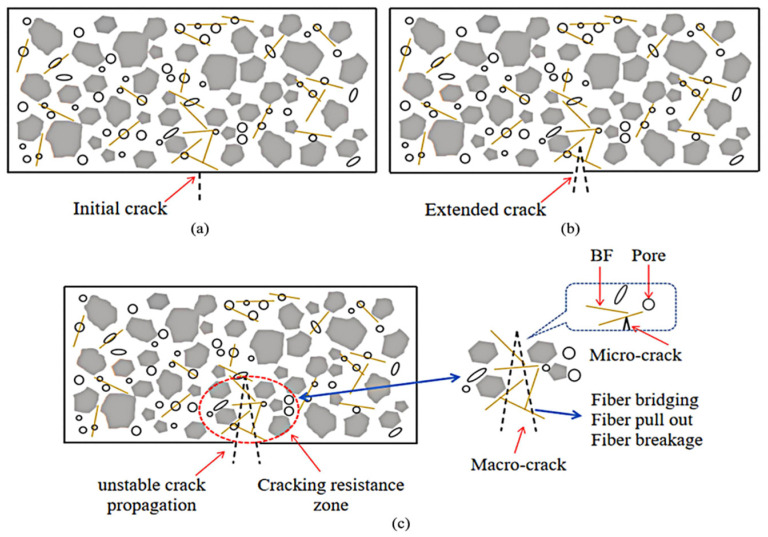
The mechanism process of basalt fiber inhibiting the crack propagation in concrete. (**a**) Crack initiation process. (**b**) Crack propagation process. (**c**) Toughening and crack resistance process [36].

As shown in Figure 5, the influence of fiber length and content on compressive strength can be interpreted through two competing mechanisms: crack bridging versus defect introduction. For a given fiber content (e.g., 0.1%), the compressive strength increased with fiber length up to 9 mm (from 27.30 MPa to 32.07 MPa) but decreased at 12 mm and above (e.g., 28.13 MPa at 24 mm). This trend suggests that fibers of moderate length (9 mm) achieve sufficient anchorage to impede micro-crack propagation without excessive agglomeration. Shorter fibers (6 mm) provide a higher fiber count per unit volume but lack the embedment length to sustain bridging stress, as indicated by their lower strength gain (29.96 MPa) [27,28,29]. Longer fibers (≥12 mm) tend to entangle during mixing, as supported by the observed reduction in workability (Section 3.1), which likely introduces localized fiber clusters and interfacial voids. Regarding fiber content, at a fixed length of 9 mm, strength peaked at 0.1% (32.07 MPa at 7 days) and declined at 0.2–0.4%, despite the expectation that more fibers would provide more bridges. This decline correlates with the increased slump loss (from 72 mm at 0.1% to 40 mm at 0.4% for 9 mm fibers), indicating that excessive fiber content impairs dispersion and matrix compactness [30,31,32]. The net compressive strength is therefore governed by a trade-off: a certain fiber dosage and length can effectively bridge cracks and delay failure, but beyond an optimum, fiber agglomeration and poor workability introduce initial defects that outweigh the bridging benefit. This competitive interpretation aligns with observations in the literature [33,34,35] but is directly supported here by the concurrent measurement of workability and strength across a range of parameters.

It is important to note that the concrete specimens in this study were cured under standard laboratory conditions (temperature 20 ± 2 °C, relative humidity ≥ 95%). This approach is commonly used for initial material characterization and performance comparison, allowing for the isolation of the fiber effect from other environmental variables. However, the actual service environment of a ship lock wall involves complex and coupled conditions, such as dry–wet cycles, freeze–thaw cycles, and sustained water pressure. The results obtained from standard curing, while indicative of potential performance, may not fully reflect the long-term in situ behavior. Future work will therefore focus on simulating these realistic service conditions, employing coupled environmental and mechanical loading regimes to further validate the durability enhancements observed in this study.

### 3.3. Impermeability Performance

**Figure 6:**
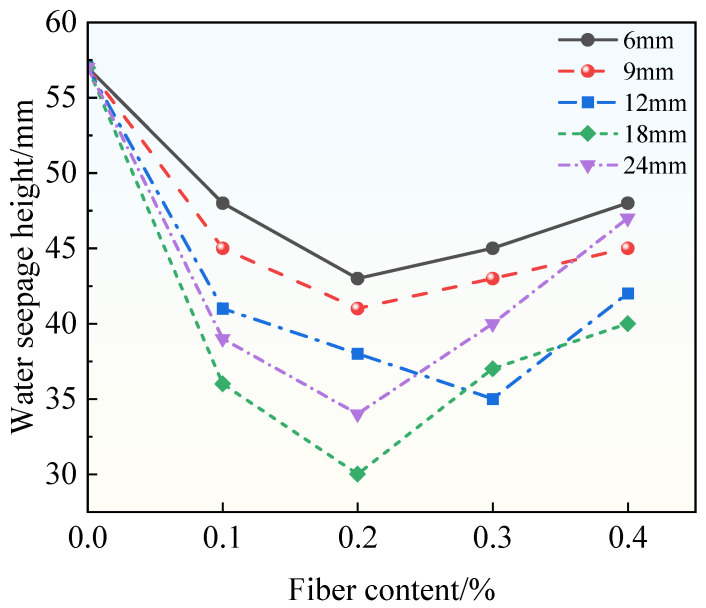
The influence of basalt fiber content and length on the height of concrete water seepage.

The durability of concrete largely depends on the penetration of harmful ions via water through its pore structure. Therefore, high resistance to water and ion permeability is essential for long-term concrete durability. In this experiment, the water penetration resistance of basalt fiber-reinforced concrete was evaluated after 28 days, and the results are shown in Figure 6.

As presented in Figure 6, plain concrete exhibited a water penetration depth of 57 mm. When basalt fiber was added, the water penetration depth was less than in the reference group, meaning the concrete became less permeable.

When the length of basalt fiber stayed the same, as more basalt fiber was added, the depth to which water seeped into the concrete first went down and then went up. When the fiber was 18 mm long and made up 0.1% and 0.2% of the concrete, water seeped 36 mm and 30 mm deep respectively, which was 36.84% and 47.37% less than in the reference concrete. When the fiber made up 0.3% and 0.4% of the concrete, water seeped 37 mm and 40 mm deep respectively, which was 35.09% and 29.82% less than in the reference concrete. Even though adding more fiber made the concrete a bit less resistant to water at these higher amounts, it was still better than the reference concrete. This shows that adding the right amount of basalt fiber can greatly improve concrete’s resistance to water, with a fiber content of 0.2% yielding the lowest water penetration depth among the tested contents.

This effect can be attributed to the influence of fibers on the water penetration resistance of concrete: when making concrete samples, adding basalt fibers helped stop coarse and fine aggregates from settling too much, which reduced problems like concrete separating into layers or water rising to the surface. As a result, the bond between aggregates and cement paste improved, and the concrete became less porous and more compact, all of which made it better at keeping water out. The fibers also broke up connected pores and created a network that made it harder for water to travel through, further improving water resistance [37,38,39].

Excessive fiber content brings about two key problems. For one, fiber agglomeration takes place, turning fibers from a functional spatial network into weak areas. Interface defects then allow solution to penetrate more easily, forming “shortcuts” that undermine impermeability. Additionally, an overabundance of fibers severely hampers concrete’s workability and dispersibility, disrupting the forming process and ultimately weakening the concrete’s durability. In short, adding too many fibers reduces concrete’s impermeability [40,41,42].

When the amount of basalt fiber stayed the same, the depth to which water seeped into the concrete first went down and then went up as the basalt fiber got longer. When the fiber amount was 0.2% and the fibers were 6 mm, 9 mm, 12 mm, and 18 mm long, the water seeped 43 mm, 41 mm, 38 mm, and 30 mm into the concrete, respectively. These depths were 24.56%, 28.07%, 33.33%, and 47.37% less than the reference group. But when the fiber length was increased to 24 mm, the water seeped 34 mm in, which was 40.35% less than the reference group. Even though the concrete was a bit less waterproof with these longer fibers, it was still better than the reference concrete. Among the tested lengths, 18 mm resulted in the smallest water penetration depth.

Too much fiber length was found to make concrete less impermeable. When the fiber length was within a suitable range, the fibers inside the concrete formed a denser spatial network structure, which allowed shrinkage stress to be distributed better and had a greater impact on connected pores and penetration paths, greatly improving the impermeability of the concrete [43,44,45]. But when the fiber length was too long, the fibers would tangle and clump together. Also, overly long fibers made the concrete harder to work with and less evenly dispersed, inevitably creating more flaws. All these factors were found to be reasons why the concrete’s impermeability decreased [46,47,48].

In this experiment, the lowest water penetration depth was observed for concrete with 0.2% fiber content and 18 mm fiber length.

### 3.4. Frost Resistance Performance

**Figure 7:**
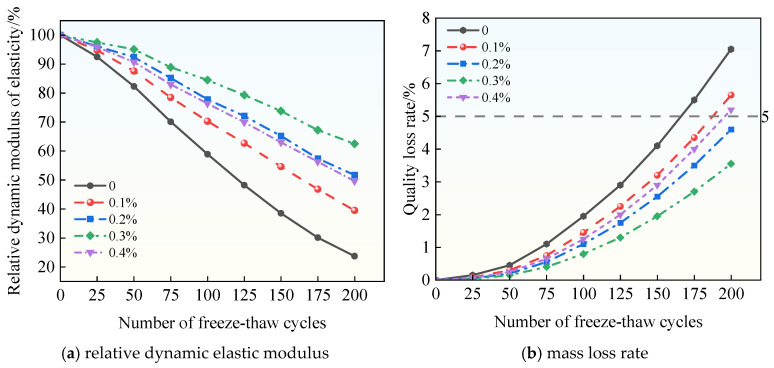
Test results of the relative dynamic elastic modulus and mass loss rate of concrete with different amounts of basalt fiber.

Based on the preceding impermeability test results, basalt fibers of 18 mm length were identified as optimal and were selected for further frost resistance evaluation. So, we chose these fibers to study how well the concrete holds up against frost. This study tested concrete samples with different amounts of basalt fibers by putting them through freeze–thaw cycles. After a varying number of these cycles, we measured how much the concrete’s dynamic elastic modulus changed relative to its original value and how much mass it lost. The results are shown in Figure 7.

As shown in Figure 7, the relative dynamic elastic modulus of all concrete specimens decreased progressively with an increasing number of freeze–thaw cycles, and the speed of this decrease varied a lot. For samples without any basalt fiber (0% content), after 75 freeze–thaw cycles, the relative dynamic elastic modulus dropped to just 70.1%. After 100 cycles, it fell to 58.9%, which no longer met the required standards. For samples with 0.1%, 0.2%, and 0.4% basalt fiber, after 175 freeze–thaw cycles, the relative dynamic elastic modulus was only 46.8%, 57.4%, and 56.3% respectively, all failing to meet the standards. However, for samples with 0.3% basalt fiber, after 200 freeze–thaw cycles, the relative dynamic elastic modulus was still at 62.5%, which still met the standards. Also, as more basalt fiber was added to the concrete, the amount by which the relative dynamic elastic modulus decreased first got smaller and then got larger. These findings suggest that adding basalt fibers can improve the concrete’s ability to withstand frost damage. This improvement is because the basalt fibers help bind the concrete together locally, protecting its internal structure from damage caused by freeze–thaw cycles. But if too many basalt fibers are added, the improvement in frost resistance becomes less effective.

Regarding mass loss, the results in Figure 7 indicate that after 100 freeze–thaw cycles, concrete with 0.1%, 0.2%, 0.3%, and 0.4% fiber had relatively small mass losses of 1.45%, 1.10%, 0.80%, and 1.25% respectively, while concrete without fibers lost significantly more mass at 1.95%. By 175 freeze–thaw cycles, the reference group (concrete without fibers) had a mass loss of 5.50%, but all fiber-reinforced concrete had mass losses below 5%. After 200 freeze–thaw cycles, only concrete with 0.2% and 0.3% fiber kept mass losses below 5%, at 4.60% and 3.55% respectively, while all other groups lost more than 5%. As more basalt fiber was added to the concrete, the reduction in mass loss rate first became smaller and then increased. However, adding too many basalt fibers reduced the improvement in frost resistance durability of the concrete [49,50,51].

The factors influencing concrete’s freeze–thaw resilience can be categorized into two groups: external conditions like temperature, humidity, duration, and the number of freeze–thaw cycles, and internal traits inherent to the concrete itself, such as ultimate tensile strain, toughness, air content, and fiber inclusion. Focusing on internal enhancements, basalt fibers, when randomly dispersed in three dimensions within the concrete matrix, interlock to form a supportive framework for the aggregates. This arrangement effectively blocks the formation of interconnected cracks before the concrete hardens and hinders the development of linked capillary pores. Consequently, adding basalt fibers refines the cement paste’s structure, boosting the concrete’s impermeability and enhancing its overall durability against freeze–thaw cycles [52,53,54].

**Figure 8:**
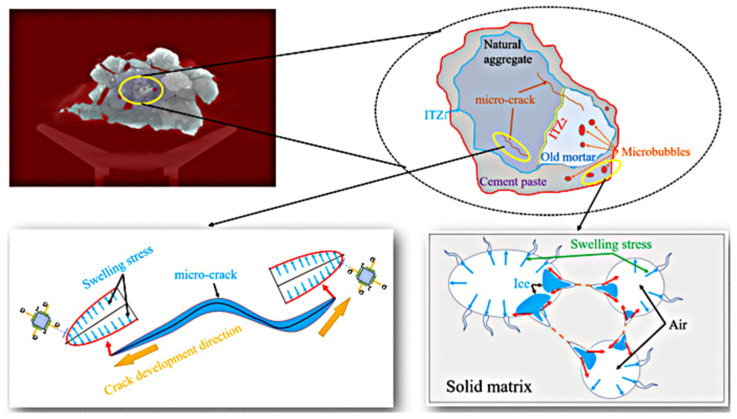
Development of internal freeze–thaw damage in the aggregate [61].

The better impermeability means outside moisture cannot easily get into the tiny holes inside the concrete, which reduces the amount of water that can freeze in those holes and makes the concrete more resistant to frost. Second, the randomly placed microfibers get tangled and overlap, which stops air from escaping when the concrete is mixed and shaped. This increases the air content in the concrete, helping to relieve the pressure caused by water and its movement during cold conditions [55,56,57]. Also, basalt fibers have a higher stiffness than the concrete itself when it first starts to set, which boosts the composite’s tensile strength during its plastic and early hardening stages. This reduces the number of tiny cracks that form naturally in the concrete, as shown in Figure 8. Finally, using the right mixing ratios and methods ensures that the fibers are evenly spread out and close enough together in the concrete, which helps it absorb more energy during freeze–thaw cycles and prevents cracking caused by frost [58,59,60].

### 3.5. Early Crack Resistance Performance

#### 3.5.1. Crack Time

**Figure 9:**
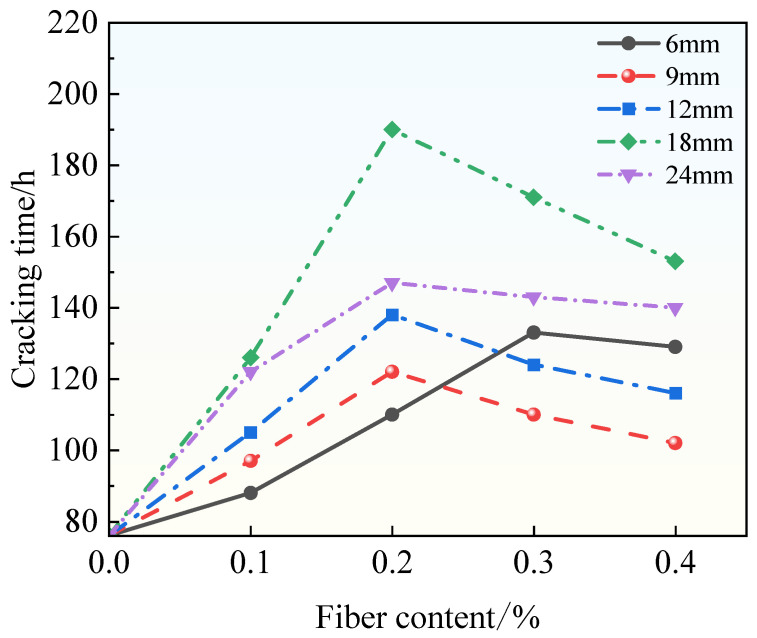
Cracking Time of Ring Specimens.

The cracking times of the circular ring specimens of each group of cement paste are shown in Figure 9.

As illustrated in Figure 9, the addition of basalt fibers to all cement paste ring specimens resulted in a delayed cracking time compared to the control group (plain concrete).

For a fixed fiber length, the cracking time initially increased and then decreased with increasing fiber content in each group of cement paste ring specimens. When the fiber was 6 mm long and made up 0.3% of the mix, cracks took 133 h to show up, which was a 75% improvement in performance. When the fiber was 9 mm long and 0.2% of the mix, cracks appeared after 122 h, showing a 60.5% improvement. With 12 mm fibers at 0.2%, cracks took 138 h, an 81.6% improvement. For 18 mm fibers at 0.2%, the longest cracking time (190 h) was observed at 0.2% fiber content and 18 mm length. Finally, with 24 mm fibers at 0.2%, cracks showed up after 147 h, reflecting a 93.4% improvement.

When the amount of fiber was the same, this study looked at cement paste ring samples with four different fiber lengths: 6 mm, 9 mm, 12 mm, and 18 mm. This study found that as the fiber length increased, the time it took for the samples to crack also increased. At every fiber amount level, the 18 mm fibers consistently gave the longest cracking time. However, when the fiber length was 24 mm, the cracking time was shorter than that of the 18 mm fibers.

The specific data on the cracking time and performance improvement of the circular ring specimens of each group of cement paste are shown in Table 6.

The table shows that adding basalt fibers greatly increased the cracking time of cement paste ring specimens in all groups. The BFRC-0.2-18 group saw the biggest increase, with cracking time improving by 150%. When looking at the average results of the five main groups, it was found that for fiber lengths between 6 and 18 mm, longer fibers led to longer cracking times and bigger improvements. However, when fiber length reached 24 mm, the improvement decreased, with cracking time being 28.9% less than that with 18 mm fibers.

#### 3.5.2. Crack Width

**Figure 10:**
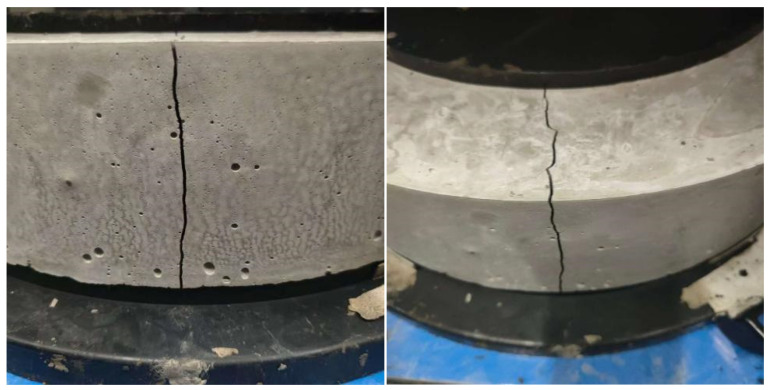
Cracking of the circular specimen.

The cracking condition of the cement paste circular ring specimen is shown in Figure 10.

**Figure 11:**
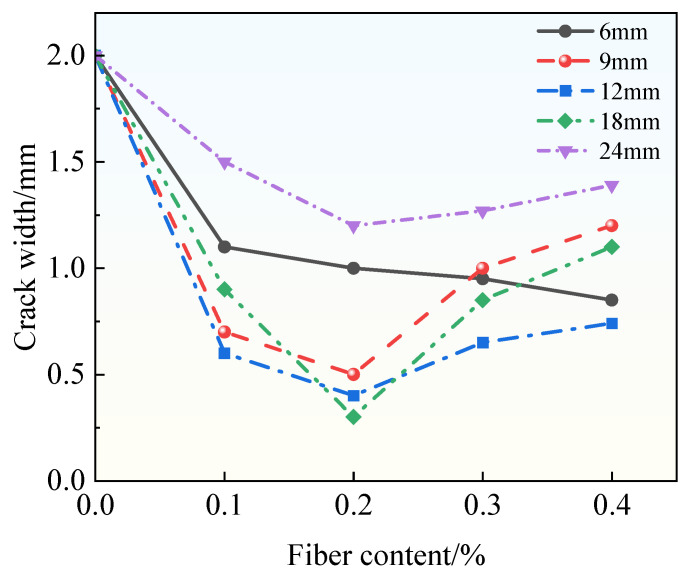
Crack Width of Circular Specimen.

The picture shows that the crack was very damaging, going all the way through the specimen from top to bottom. Upon close inspection of the cracked section, one end of a visible fiber was observed to have been pulled out, while the other end remained embedded in the cement matrix. Also, some fibers were still firmly attached at both ends to the cement. Figure 11 shows the crack widths for the circular cement paste specimens in each group.

As shown in Figure 11, the addition of basalt fibers reduced the crack width in all cement paste ring specimens compared to the samples without basalt fibers (the control group).

This study looked at how fiber content affected the crack width in cement paste ring samples, keeping the fiber length the same each time. When the fibers were 6 mm long, adding more fibers made the crack width smaller in all four groups of samples. At a fiber content of 0.4%, the crack width was 0.85 mm, which was 57.5% smaller than in the group with no fibers. When fibers used were 9 mm, 12 mm, 18 mm, or 24 mm long, the crack width in the four groups of samples first got smaller and then got bigger as we added more fibers. The smallest crack width always happened when the fiber content was 0.2%. For example, when the fibers were 18 mm long and made up 0.2% of the sample, the crack width was just 0.3 mm, the smallest value observed.

When looking at the crack widths for each group of specimens that had the same amount of fiber, we found that the only clear thing was that the crack widths got smaller to different extents. In other words, when five different fiber lengths were tested, we saw that as the amount of fiber increased, the way crack widths changed over time was different for each group.

The specific data of crack widths and performance improvement for each group of cement paste ring specimens are presented in Table 7.

When looking closely at the crack widths in cement paste ring samples, we saw that while there were some basic patterns, the data from different groups varied a lot. But when examining the results of the concrete strength tests and the cracking times from the ring restraint tests, we generally found clearer patterns. The observed variability in crack width (Table 7) can be partially attributed to the stochastic distribution of fibers within the cement paste matrix. Unlike concrete, which contains coarse aggregates that may help disperse fibers, the cement paste used in the ring test has higher viscosity and no aggregates [62,63,64]. This promotes local fiber clustering or depletion zones, as evidenced by the non-monotonic relationship between fiber content and crack width. For example, at a fiber length of 9 mm, increasing the content from 0.1% to 0.2% reduced the crack width from 0.70 mm to 0.50 mm, but further increases to 0.3% and 0.4% widened the crack to 1.00 mm and 1.20 mm, respectively. This suggests that an optimal fiber content (0.2% for 9 mm fibers) exists for crack control; beyond this point, fiber agglomeration creates stress concentration zones that act as crack initiation sites, counteracting the intended reinforcement.

Fiber length also plays a critical role. For a fixed content of 0.2%, crack width decreased progressively from 1.00 mm (6 mm) to 0.30 mm (18 mm), but increased again at 24 mm (1.20 mm). The improvement at 18 mm is consistent with longer fibers providing greater bridging across crack surfaces, requiring more energy for pull-out [65,66]. However, the reversal at 24 mm indicates that excessively long fibers are difficult to disperse uniformly in the high-viscosity cement paste, leading to entanglement and weak interfacial zones. This interpretation is supported by the corresponding cracking time data (Table 6), where the 18 mm fiber group showed the longest delay (190 h), while the 24 mm group showed a shorter delay (147 h). Thus, the crack resistance performance is governed by a balance between fiber length and dispersion quality, rather than simply “longer is better” as sometimes stated in the literature [67,68]. In this particular experiment, the cement paste with 0.2% basalt fibers that were 18 mm long showed the best resistance to cracking in the ring restraint test.

The wide variation in crack width data observed in Table 7 can be attributed to several factors inherent to the ring test and the nature of fiber-reinforced cementitious materials. Firstly, the random distribution of basalt fibers within the cement paste matrix is inherently non-uniform. While the overall fiber content is controlled, local agglomeration, entanglement, or fiber-poor regions can occur, especially given the higher viscosity of the cement paste compared to concrete. These local variations directly influence the reinforcement efficiency at the location where a crack eventually initiates. Secondly, the random initiation and propagation of cracks in the ring test mean that a crack may form in an area with a higher-than-average fiber density, leading to a very narrow crack, or in a fiber-poor zone, resulting in a wider crack. This stochastic nature of both fiber distribution and crack location contributes significantly to the observed scatter. Furthermore, the absence of additional confinement or notches in the standard ring test means that the crack path is not predetermined, adding another layer of variability. These factors collectively explain the high standard deviation in crack width measurements, even though the overall trend of improved crack resistance with optimal fiber parameters (e.g., BFRC-0.2-18) remains statistically and practically significant.

### 3.6. Results and Analysis of Abrasion Resistance Test

Based on previous performance evaluations, basalt fibers of 18 mm length were selected for abrasion resistance testing. Concrete samples with varying basalt fiber contents underwent abrasion tests, with their weights measured before and after. Following the “Test Code for Hydraulic Concrete” (SL/T 352-2020), this study calculated the wear rate and abrasion resistance strength. Detailed findings are summarized in Table 8.

As presented in Table 8, the incorporation of fibers reduced the wear rate of concrete. When the amount of fibers went up from 0.1% to 0.4%, the wear rate of the concrete dropped by 5.9%, 19.6%, 45.1%, and 11.8% compared to concrete without added fibers. The minimum wear rate (2.8%) was obtained at a fiber content of 0.3% by volume. But when there was more fiber than this, the wear rate started to go up again. The reason for this is that too much fiber can clump together inside the concrete, causing stress to build up in those areas. These clumps of fiber become weak spots in the concrete [69,70]. When high-speed steel balls hit these weak areas, cracks easily form on the concrete surface. As the steel balls keep hitting, the cracks spread outwards. The surface layer of the concrete gradually breaks off, eventually creating grooves of different depths due to wear.

As the abrasion resistance test went on, abrasion grooves made the concrete surface uneven. When high-velocity water flowed over these uneven surfaces, cavitation flows containing numerous air bubbles were generated. When these bubbles moved close to the concrete surface under high water pressure, they tended to burst. The sudden force from the bursting bubbles kept hitting the concrete surface, causing small areas of damage [71,72]. This small damage made the uneven surface even bigger, creating a cycle that led to a lot of abrasion damage on the concrete, which shortened the material’s lifespan [73,74].

**Figure 12:**
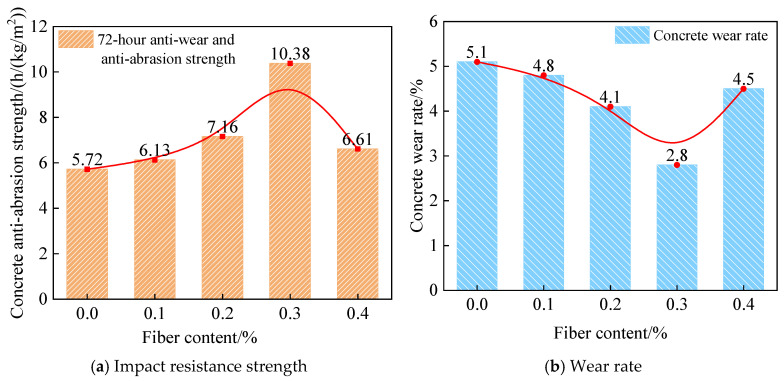
Experimental results of the anti-abrasion strength and wear rate of concrete with different amounts of basalt fiber.

After a cumulative 72 h of abrasion testing, the abrasion resistance strength and wear rate were measured, and the results are shown in Figure 12.

The picture shows that adding fibers to concrete makes it much more resistant to abrasion. As we added more fibers, the concrete’s abrasion resistance first went up and then went down. When the fiber content rose from 0.1% to 0.4%, the abrasion resistance of the concrete improved by 7.2%, 25.2%, 81.5%, and 15.6% respectively compared to concrete without added fibers. The highest abrasion resistance value (10.38 h/(kg/m^2^)) was recorded at a fiber content of 0.3%. At 0.4% fiber content, the abrasion resistance dropped significantly but was still 15.6% better than the concrete without fibers. The reason fibers improve abrasion resistance can be explained by looking at how energy is transferred: steel balls driven by fast-moving water have a lot of kinetic energy. When these balls hit the concrete surface and bounce back, some of this energy is absorbed by the concrete’s elastic and plastic deformation. As plastic deformation increases, particles on the concrete’s surface start to come off. The rough surface left by these missing particles makes abrasion damage worse [44,75,76].

This happened because the right amount of fibers was spread randomly and evenly throughout the concrete, creating a special three-dimensional network. The fibers stuck to and rubbed against the cement mixture, absorbing some of the energy from movement and preventing cracks from starting and spreading when the concrete was under stress. This made the concrete much tougher and stopped pieces from breaking off. The fibers also helped the concrete resist being hit or worn away, reducing the amount of permanent deformation caused by steel balls pressing on its surface [77,78].

### 3.7. Microstructural Observation by SEM

**Figure 13:**
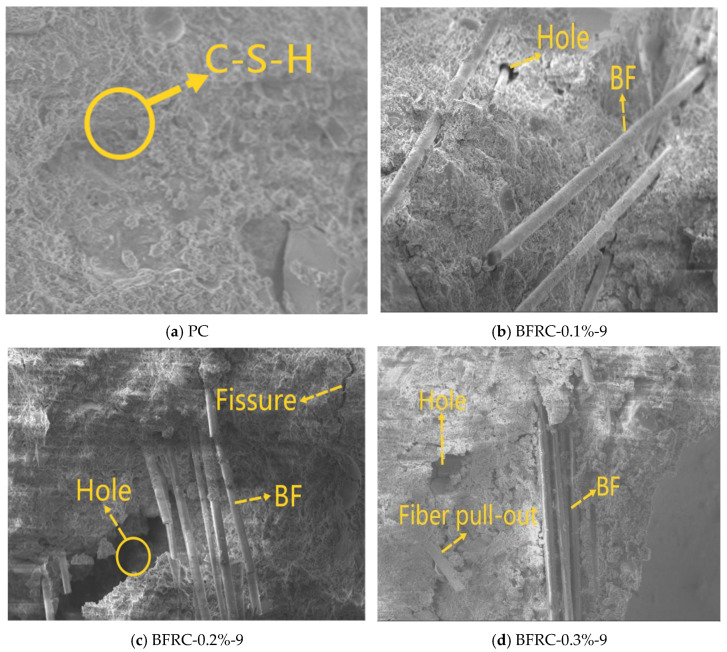
Microstructure diagram of BFRC with different doping amounts but a BF length of 9 mm.

The SEM images in Figure 13 provide morphological evidence for the competing mechanisms discussed in Section 3.2. In plain concrete (Figure 13a), a porous microstructure with interconnected cracks is visible, consistent with its lower compressive strength. At 0.1% fiber content (Figure 13b), the matrix appears relatively denser, with fewer observable pores. Notably, fibers are individually dispersed, and the fiber–matrix interface shows small surface irregularities that may facilitate mechanical interlocking. No chemical bonding is evident (i.e., no reaction rim or elemental diffusion), indicating that reinforcement is primarily physical. At 0.2% fiber content (Figure 13c), fiber clusters begin to appear, and localized voids are observed around these clusters. This corresponds to the onset of workability loss (slump reduced from 72 mm at 0.1% to 60 mm at 0.2% for 9 mm fibers). At 0.3% (Figure 13d), extensive fiber agglomeration is seen, with large gaps between fiber bundles and the matrix. These gaps serve as stress concentrators and crack initiation sites, explaining why compressive strength declined beyond 0.1% despite more fibers being present [79,80].

Thus, the SEM observations directly illustrate the trade-off: a low fiber content (0.1%) enables relatively uniform dispersion and moderate bridging, while higher contents (>0.2%) introduce agglomeration-induced defects that undermine mechanical performance. This morphological–mechanical correlation is a key contribution of this study, albeit limited to qualitative assessment due to the absence of EDS and porosity quantification [57,81].

In the absence of basalt fibers, the interface between cement paste and coarse aggregate appeared well-bonded and relatively dense under SEM observation. In Figure 13b, the concrete paste had fewer tiny holes and cracks, showing it was fairly dense. In Figure 13c, the paste had a bit more holes and cracks than when 0.1% BF was added. In Figure 13d, there were far more holes in the paste compared to the other three amounts of BF, and there were also bigger gaps between the BF and the paste. This mainly happened because adding more fibers made the concrete much harder to work with and less fluid. The mechanical properties of concrete under slow, steady force are directly linked to how the failure interface behaves. When a certain amount of BF was added, during compressive failure, a clear pull-out behavior occurred at the failure interface, which greatly boosted the concrete’s compressive strength. However, since BF was added to hydraulic concrete, it created some holes and cracks at the failure interface, making it weaker. As a result, the compressive and tensile strengths of basalt fiber-reinforced concrete (BFRC) were relatively lower [82]. The influence of basalt fibers on compressive strength can be attributed to these two competing effects. However, quantitative validation (e.g., via EDS or porosity analysis) would be required to fully establish the underlying mechanisms.

**Figure 14:**
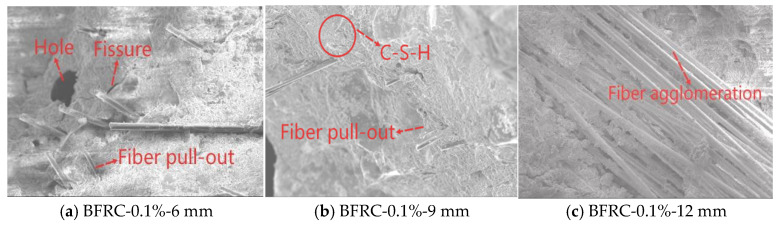
Microstructure diagram of BFRC with a BF content of 0.1% but different lengths.

Figure 14 shows SEM micrographs of BFRC with a fixed BF content of 0.1% but different lengths. In Figure 14a, some pores were seen on the mortar surface. When the BF was 9 mm long, there were far fewer pores on the mortar surface than when it was 6 mm long. When the BF was 12 mm long, the bond between the mortar and BF became noticeably weaker. There were pores in the mortar, and cracks were also seen around the BF. When comparing Figure 14b,c, it was found that the 9 mm-long fibers were spread out more evenly in the cement mortar than the 12 mm-long ones. Figure 14c shows that a bunch of fibers stuck together, making the structure in the interfacial zone looser and reducing the overall strength of the concrete.

The underlying causes of certain phenomena were thoroughly examined. Initially, basalt fibers possess a natural “clustering” propensity, which means that even after thorough mixing, some fibers tend to clump together. Secondly, the excessive length of these fibers hindered their complete and uniform dispersion within the designated mixing period [83,84,85]. The dual influence of basalt fibers’ crack-resisting capabilities and their relatively weak interaction with the concrete matrix resulted in a moderate enhancement of compressive strength when fibers were added, underscoring the necessity of precise fiber content control [18,57]. Concrete containing 0.1% fibers of 9 mm length exhibited the highest compressive strength, suggesting consistency between microstructural observations and macroscopic test results.

### 3.8. Trade-Offs in Fiber Parameter Selection

The experimental results indicate that the optimal fiber parameters differ depending on the performance metric of interest. For compressive strength, the highest values were recorded at a fiber content of 0.1% and length of 9 mm. For crack resistance (delayed cracking and reduced crack width), a higher content (0.2%) and longer fiber (18 mm) proved more effective. Impermeability also favored 0.2% and 18 mm, while abrasion resistance peaked at 0.3% (with 18 mm length). Freeze–thaw resistance was best at 0.3% fiber content.

These variations suggest that no single fiber parameter combination simultaneously optimizes all properties. In engineering practice, the selection should prioritize the most critical requirements for the specific application. For ship lock walls, where early-age cracking and long-term impermeability are primary concerns, a fiber content of 0.2% and length of 18 mm is recommended. If compressive strength is the main design criterion, 0.1% and 9 mm fibers would be more suitable.

It should be noted that this study did not perform a formal multi-objective optimization (e.g., response surface methodology or genetic algorithm). The recommended values are based on direct comparison of test results within the selected parameter ranges. Future work could employ optimization techniques to identify global optimum combinations considering multiple performance indices simultaneously.

## 4. Conclusions

This study experimentally investigated the effects of basalt fiber content and length on the workability and mechanical strength of hydraulic concrete. Scanning electron microscopy was also used to examine the microstructure of basalt fiber-reinforced concrete and to elucidate the fiber-induced microstructural changes. The main findings are summarized as follows:

The workability of basalt fiber-reinforced concrete decreased with increasing fiber length and content. At a fiber length of 24 mm and a content of 0.4%, the slump decreased by 75%. However, when the fiber amount was more than 0.1% and the length was longer than 24 mm, this worsening slowed down and eventually stopped changing.

Among the tested parameters, a fiber content of 0.1% and a length of 9 mm yielded the highest compressive strength gains. However, considering workability and crack resistance, a fiber content of 0.2% and length of 18 mm is recommended for applications requiring enhanced durability.

When the amount of fiber stayed the same, we saw that in all groups, the longer the fibers were, the longer it took for the cement paste ring samples to crack, and this improvement got bigger over time. The best improvement—150.0%—happened when the basalt fiber amount was 0.2% and the fiber length was 18 mm.

Durability performance was significantly enhanced by the incorporation of basalt fibers. The optimal impermeability was achieved with a 0.2% fiber content and 18 mm length, reducing the water penetration depth by 47.37% compared to plain concrete. For freeze–thaw resistance, a 0.3% fiber content maintained the relative dynamic elastic modulus above the standard threshold (62.5%) even after 200 freeze–thaw cycles. Regarding abrasion resistance, a 0.3% fiber content resulted in the lowest wear rate (2.8%) and the highest impact resistance strength (10.38 h/(kg/m^2^)), representing a 45.1% reduction in wear and an 81.5% increase in strength compared to the reference group.

SEM observations indicate that uniformly dispersed basalt fibers may refine the pore structure and inhibit micro-crack propagation through a bridging effect, while excessive fiber content or length leads to agglomeration and interfacial defects. It should be noted that these SEM results are morphological in nature; no EDS or quantitative porosity analysis was conducted. Future work incorporating these techniques is necessary to fully elucidate the reinforcement mechanisms.

It should also be noted that the results and recommendations presented in this study are based on a single concrete mix design (C30 grade, water-binder ratio 0.40, with fly ash) and a specific set of basalt fiber parameters (lengths 6–24 mm, dosages 0.1–0.4%). Therefore, the findings may not be directly generalizable to other mix proportions, fiber types, or service conditions. Further validation with a wider range of materials and mix designs is recommended.

## Figures and Tables

**Table 1:** Main Physical Properties of Cement.

Test Item	Compressive Strength (MPa)		Bending Strength (MPa)		Setting Time (min)		Consistence (%)	Stability (mm)
	3 d	28 d	3 d	28 d	Initial Set	Final Set		
Test cement	26.6	50.7	5.2	8.6	153	293	26.0	1.8
standard request	≥17.0	≥42.5	3.5	6.5	≥45 min	≤600	/	≤5.0

**Table 2:** Main Technical Indicators of Fly Ash.

Performance Index	Measured Value	Standard Specified Value (II)
Fineness (percentage of residue after passing through a 45 μm square hole sieve) (%)	15.2	≤25.0
water demand ratio (%)	95	≤105.0
loss on ignition (%)	2.8	≤8.0
moisture content (%)	0.2	≤1.0
Stability (mm)	1.6	≤5.0

**Table 3:** Performance Index Parameters of Basalt Fiber.

Diameter of a Single Filament/μm	Density/(g/cm^3^)	Elasticity Modulus/GPa	Tensile Strength/MPa	Elongation at Break/%
7–15	2.63–2.65	91–110	3000–4800	2.5

**Table 4:** Mix Ratio of Basalt Fiber Concrete (kg/m^3^).

Serial Number	Water	Cement	Coal Ash	River Sand	Macadam	Water Reducer	Basalt Fiber
PC	180	360	90	700	1050	3.6	0
BFRC-0.1%	180	360	90	700	1047.62	3.6	2.38
BFRC-0.2%	180	360	90	700	1045.23	3.6	4.77
BFRC-0.3%	180	360	90	700	1042.85	3.6	7.15
BFRC-0.4%	180	360	90	700	1040.47	3.6	9.53

Note: The number “PC” indicates plain concrete; “BFRC-0.1%” represents basalt fiber content of 0.1%, and so on. The fiber lengths varied as 6, 9, 12, 18, and 24 mm. For each length, the mix proportions followed the same pattern as shown for BFRC-0.1% to BFRC-0.4%.

**Table 5:** Slump values (mm) of basalt fiber-reinforced concrete.

Fiber Length (mm)	Fiber Content (% by Volume)			
	0.1	0.2	0.3	0.4
0 (PC)	110	110	110	110
6	97	82	50	44
9	72	60	56	40
12	58	54	38	31
18	42	38	32	30
24	40	31	30	27

**Table 6:** Summary of cracking time for each group of circular specimens.

Group Indication	Cracking Time (h)	Performance Improvement	
PC	76		
BFRC-0.1-6	88	15.8%	51.3%
BFRC-0.2-6	110	44.7%	
BFRC-0.3-6	133	75.0%	
BFRC-0.4-6	129	69.7%	
BFRC-0.1-9	97	27.6%	41.8%
BFRC-0.2-9	122	60.5%	
BFRC-0.3-9	110	44.7%	
BFRC-0.4-9	102	34.2%	
BFRC-0.1-12	105	38.2%	58.9%
BFRC-0.2-12	138	81.6%	
BFRC-0.3-12	124	63.2%	
BFRC-0.4-12	116	52.6%	
BFRC-0.1-18	126	65.8%	110.5%
BFRC-0.2-18	190	150.0%	
BFRC-0.3-18	171	125.0%	
BFRC-0.4-18	153	101.3%	
BFRC-0.1-24	122	60.5%	81.6%
BFRC-0.2-24	147	93.4%	
BFRC-0.3-24	143	88.2%	
BFRC-0.4-24	140	84.2%	

**Table 7:** Summary of crack opening widths of circular specimens in each group.

Group Indication	Crack Width (mm)	Falling Range	
PC	2.00		
BFRC-0.1-6	1.10	45.0%	51.3%
BFRC-0.2-6	1.00	50.0%	
BFRC-0.3-6	0.95	52.5%	
BFRC-0.4-6	0.85	57.5%	
BFRC-0.1-9	0.70	65.0%	57.5%
BFRC-0.2-9	0.50	75.0%	
BFRC-0.3-9	1.00	50.0%	
BFRC-0.4-9	1.20	40.0%	
BFRC-0.1-12	0.60	70.0%	70.1%
BFRC-0.2-12	0.40	80.0%	
BFRC-0.3-12	0.65	67.5%	
BFRC-0.4-12	0.74	63.0%	
BFRC-0.1-18	0.90	55.0%	60.6%
BFRC-0.2-18	0.30	85.0%	
BFRC-0.3-18	0.85	57.5%	
BFRC-0.4-18	1.10	45.0%	
BFRC-0.1-24	1.50	25.0%	33.0%
BFRC-0.2-24	1.20	40.0%	
BFRC-0.3-24	1.27	36.5%	
BFRC-0.4-24	1.39	30.5%	

**Table 8:** Wear rate and anti-abrasion strength of fiber-reinforced concrete with different fiber contents.

Fiber Content (%)	Pre-Processing Quality (kg)	Post-Polishing Quality (kg)	Quality Loss (kg)	Wear Rate (%)	Impact Resistance Strength (h/(kg/m^2^))
0	17.55	16.66	0.89	5.1	5.72
0.1	17.37	16.54	0.83	4.8	6.13
0.2	17.49	16.78	0.71	4.1	7.16
0.3	17.69	17.2	0.49	2.8	10.38
0.4	17.28	16.51	0.77	4.5	6.61

## Data Availability

The original contributions presented in this study are included in the article. Further inquiries can be directed to the corresponding author.
